# Design of Polymer Nanodielectrics for Capacitive Energy Storage

**DOI:** 10.3390/nano13172394

**Published:** 2023-08-22

**Authors:** Prajakta Prabhune, Yigitcan Comlek, Abhishek Shandilya, Ravishankar Sundararaman, Linda S. Schadler, Lynda Catherine Brinson, Wei Chen

**Affiliations:** 1 Thomas Lord Department of Mechanical Engineering and Material Science, Duke University, Durham, NC 27708, USA; prajakta.prabhune@duke.edu (P.P.); cate.brinson@duke.edu (L.C.B.); 2 Department of Mechanical Engineering, Northwestern University, Evanston, IL 60208, USA; yigitcancomlek2024@u.northwestern.edu; 3 Materials Science and Engineering, Rensselaer Polytechnic Institute, Troy, NY 12180, USA; abhishandy@protonmail.com (A.S.); sundar@rpi.edu (R.S.); 4 College of Engineering and Mathematical Sciences, University of Vermont, Burlington, VT 05405, USA; linda.schadler@uvm.edu

**Keywords:** polymer nanodielectrics, capacitive stored energy, breakdown strength, extrinsic interface, intrinsic interface, trap depth, finite difference simulations, latent variable gaussian process, Bayesian optimization, global sensitivity analysis

## Abstract

Polymer nanodielectrics present a particularly challenging materials design problem for capacitive energy storage applications like polymer film capacitors. High permittivity and breakdown strength are needed to achieve high energy density and loss must be low. Strategies that increase permittivity tend to decrease the breakdown strength and increase loss. We hypothesize that a parameter space exists for fillers of modest aspect ratio functionalized with charge-trapping molecules that results in an increase in permittivity and breakdown strength simultaneously, while limiting increases in loss. In this work, we explore this parameter space, using physics-based, multiscale 3D dielectric property simulations, mixed-variable machine learning and Bayesian optimization to identify the compositions and morphologies which lead to the optimization of these competing properties. We employ first principle-based calculations for interface trap densities which are further used in breakdown strength calculations. For permittivity and loss calculations, we use continuum scale modelling and finite difference solution of Poisson’s equation for steady-state currents. We propose a design framework for optimizing multiple properties by tuning design variables including the microstructure and interface properties. Finally, we employ mixed-variable global sensitivity analysis to understand the complex interplay between four continuous microstructural and two categorical interface choices to extract further physical knowledge on the design of nanodielectrics.

## 1. Introduction

For capacitive energy storage applications, it is desirable to maintain low loss $\left(\varepsilon_{r}^{\prime \prime }\right)$, while having high dielectric constant $\left(\varepsilon_{r}^{\prime }\right)$ and high breakdown strength $\left(E_{b}\right)$ to obtain a high energy density $\left(U_{d}\right)$, which is also referred to as stored energy density (SED),

(1) $$U_{d}=\frac{1}{2}\varepsilon_{0}\varepsilon_{r}^{\prime }E_{b}^{2}.$$

Polymer nanodielectrics are very well suited for this purpose as they can combine high permittivity nanosized metal oxides particles with high breakdown strength polymers [1]. Experimental combinations at a range of volume fractions, particle functionalization, particle permittivity, and particle size and shapes have been explored and indicate a complex interplay between the properties of interest [2,3,4]. An increase in aspect ratio leads to field concentrations in the polymer and hence can further increase the permittivity [5]. At the same time, these field concentrations can decrease breakdown strength and increase losses [6]. Functionalization of the nanoparticles using voltage stabilizing/carrier trapping molecules can enhance breakdown strength [7,8]. However, both these strategies can impact interfacial polarization and can increase the loss [9]. Conducting and magnetic nanoparticles, through better electric polarization characteristic, can improve permittivity but may create a percolation network leading to leakage currents, again leading to high losses and low breakdown strength [10,11,12,13,14]. Overall, the results to date indicate that while one property of the system can be improved, there is typically an adverse effect on the other properties.

Another key aspect that greatly contributes to nanocomposite behavior is the properties of the interfacial region [15], which we call the interface in this paper. In addition to being able to create an extrinsic interfacial region through the functionalization of nanoparticles, local property measurements have shown the existence of an intrinsic interfacial region with significantly changed material properties such as glass transition temperature, elastic modulus, and permittivity due to changes in chain mobility, crosslink density, or crystallinity [16,17]. It is well-established that properties and/or percolation of the interface significantly influence nanocomposite properties [18,19] and hence must be considered in the design approach. Past works have used an inverse fitting approach to bulk properties to elucidate the local interface properties [20,21,22,23].

Combinations of extrinsic and intrinsic interface designs have been probed experimentally, by grafting nanoparticles with both short and long-chain molecules. Bimodal brushes consisting of short voltage stabilizing and a low graft density of long polymer chains have been used to achieve control over both dispersion and breakdown strength [24,25,26,27]. There have been only a few reported cases, however, of increases in both permittivity and breakdown strength. In those situations, the literature suggests that the charge-trapping ability of the interface is what allows that unusual combination of properties [7,8]. In this work, we hypothesize that we can increase the energy storage by using a modest aspect ratio filler to increase the permittivity and mitigate the accompanying reduction in dielectric breakdown strength by creating an extrinsic interface of charge-trapping ligands on the particle surface [28,29,30]. Additionally, increases in loss could be mitigated by tailoring the intrinsic interface [31].

Testing this hypothesis involves exploring a highly multidimensional design space for probing properties at multiple different scales, namely first-principles calculations for trap densities at interfaces, Monte Carlo for carrier mobility prediction and continuum scale simulated permittivity-loss. This renders the polymer nanodielectrics design problem difficult to handle solely with experiments or computational methods. Recently a great deal of effort has been directed toward tackling materials design problems of such complexity by combining experimental characterization, physics-based multiscale computational models, and predictive modelling using machine learning (ML) methods [32,33]. Existing computational designs are predominantly performed on 2D models and do not consider the important role of various modes of interfacial design. Similarly, our previous work provided a perspective on how multiple aspects of such a data-centric design process of polymer nanodielectrics come together and interact with each other [34]. A case study based on octyl silane-modified silica nanoparticles in a polystyrene matrix was used to demonstrate the steps involved in the design approach. The framework was then adapted to design 2D spherical nanoparticles with a designated choice of constituents like the matrix polymer and the particle functionalization chemistry [34,35].

To search for the above hypothesized optimal property niche in the wider multidimensional design space proposed here, we build on our prior work in designing nanodielectrics and use a modern computational design framework coupled with multiscale, physics-based, 3D dielectric modelling and simulation methods [36]. Precisely, we propose a design framework that tailors the microstructure (volume fraction (VF), aspect ratio (AR), dispersion, and isotropy of nanofillers) and interface (extrinsic and intrinsic) choices to identify the materials designs that optimize the conflicting dielectric loss and stored energy density properties. We use three conducting polymer ligands as extrinsic interface options and employ four specific intrinsic interfaces that encompass differing degrees of loss and permittivity relative to the neat matrix. These choices are compiled based on existing work [20,23,24,25,26,27,29,30,31]. The first principle calculation of trap states corresponding to each extrinsic molecule choice is performed and fed to Monte Carlo simulations of carrier mobility. Prediction of breakdown strength from carrier mobility is based on an existing calibration [34].

The four numerical microstructural and two interface choices constitute a mixed-variable design space. As a result, the overall design framework exhibits a significant advancement over any existing experimental study in terms of the number of design parameters. Specifically, the design framework employs a large degree of freedom 3D finite difference simulation strategy to evaluate nanodielectric properties of the complex anisotropic microstructures, the novel Latent Variable Gaussian Process (LVGP) ML model to learn the relationship between the mixed-variable design space and properties, and Bayesian optimization (BO) to identify the novel nanodielectric designs. Finally, an important highlight of this study is the implementation of the mixed-variable metamodel-based global sensitivity analysis to gain and extract physical insights into the collective relation between nanodielectric design variable properties.

The organization of this paper is as follows. First, Section 2 provides details regarding the nanodielectrics generation, physics-based 3D dielectric property evaluation, the machine learning and Bayesian optimization-based design framework, and the global sensitivity analysis methods. Next, we present the solutions obtained from the design optimization and global sensitivity analysis in Section 3 and we conclude our paper with a discussion of the results and the conclusions drawn from our research in Section 4.

## 2. Materials and Methods

This section describes the material system of interest and further details on the material design space, simulation methods for calculating dielectric properties (permittivity, loss, breakdown strength, and stored energy density), the design optimization framework, and the global sensitivity analysis.

### 2.1. The Mixed-Variable Nanodielectrics Design Space

Due to their complex structure, the design of nanodielectric materials requires special attention. The design space of these materials contains multiple physical phenomena integrated together. Specifically, a tailored microstructure and carefully selected interfacial layers are required to design the material system for capacitor applications. Nanodielectric morphology can be characterized by the four unique physical filler descriptors for microstructure and two interfacial layer choices shown in Table 1. Here, the microstructural variables are represented in a quantitative space whereas the interfacial variables are represented with qualitative choices, making the design space of nanodielectrics a mixed-variable one. The bounds of the design space values were drawn from past experimental data and interfacial properties analysis [20,23,24,25,26,27,29,30,31].

### 2.2. The Design Framework

**Figure 1:**
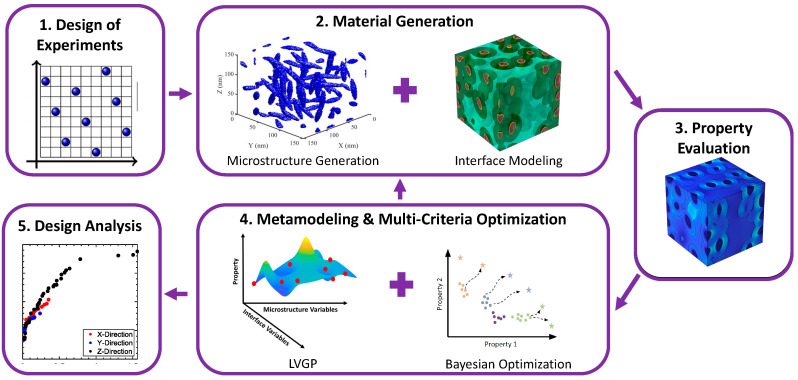
The mixed-variable nanodielectrics design framework.

The nanodielectrics design optimization framework consists of multiple stages integrated together as shown in Figure 1. First, to learn the relationships between design variables and properties, a mixed-variable initial design of experiments is created (Box 1 and Table 1). Next, microstructures characterized by different physical descriptors and interface choices are reconstructed using the methodology described in Section 2.2.1 and Section 2.2.2 (Box 2). Reconstructed images are then passed on to the Property Evaluation stage to evaluate the dielectric loss and stored energy density (SED) properties of the given nanodielectric designs (Box 3, Section 2.2.3). Next, the Latent Variable Gaussian Process (LVGP) machine learning model is trained to learn the relationships between the design variables and the two properties. Then, a well-known metamodel-based multi-objective optimization algorithm, Bayesian optimization, is implemented to identify the nanodielectric designs that optimize both properties (Box 4, Section 2.2.4). Finally, the novel designs identified by the framework are analyzed further to extract knowledge regarding the design of nanodielectrics (Box 5, Section 2.2.5).

#### 2.2.1. Design of Experiments

To initialize the design optimization framework, we created a mixed-variable design of experiments (DOE) using sliced-optimal Latin hypercube sampling with a specified number of samples and variables based on Table 1 [37]. The sliced-optimum Latin hypercube sampling is an extension of orthogonal sampling where optimum Latin hypercube sampling is implemented to sample the initial space and the space is then divided into orthogonal arrays to account for the qualitative (categorical) design variables in the design space. Specifically, an optimal Latin hypercube space is created and for each qualitative variable, the design space was sliced into $p_{i}$ sections, where $p_{i}$ represents the number of unique options for each qualitative variable. Each DOE design is assigned to a qualitative variable that falls under the sliced section. Compared to other sampling methodologies such as orthogonal or random sampling, our DOE approach excels in providing optimum initial design points that are sampled comprehensively throughout the design space for mixed-variable (qualitative and quantitative) design optimization applications. As a result, this approach enables us to select initial nanodielectric designs that cover the design space as evenly as possible for sufficient model fitting.

#### 2.2.2. Material Generation

##### Microstructure Characterization and Reconstruction

Microstructure Characterization and Reconstruction (MCR) is essential for extracting critical microstructure features and creating statistically equivalent microstructures that serve as inputs to structure-property evaluations. Inspired by existing experimental images and previous work, we decided to characterize the complex microstructure of nanodielectric materials with physical descriptors. Specifically, we determined that the volume fraction (VF), aspect ratio (AR), dispersion, and orientation of fillers (isotropy) are the main characteristics of these materials that differentiate one from another on the microstructural level [35,36]. The Orientation Variation (OV) describes the isotropy of the system by the variation of a normal distribution that assigns specific orientation to fillers. The orientation distribution is generated through $\theta =N\left(\pi /2,OV*\pi /2\right)$, where θ is a vector of angles assigned to individual fillers. Here, OV of 0 generates a vector of angles where all fillers are aligned in the Z-direction, making the structure anisotropic. OV of 1 generates a distribution of angles with a mean of π/2 and a variance of π/2, leading to an isotropic structure. Overall, these four defined physical descriptors can uniquely characterize each nanodielectric material and provide the necessary information to reconstruct these materials systems with tailored characteristics for property optimization.

**Figure 2:**
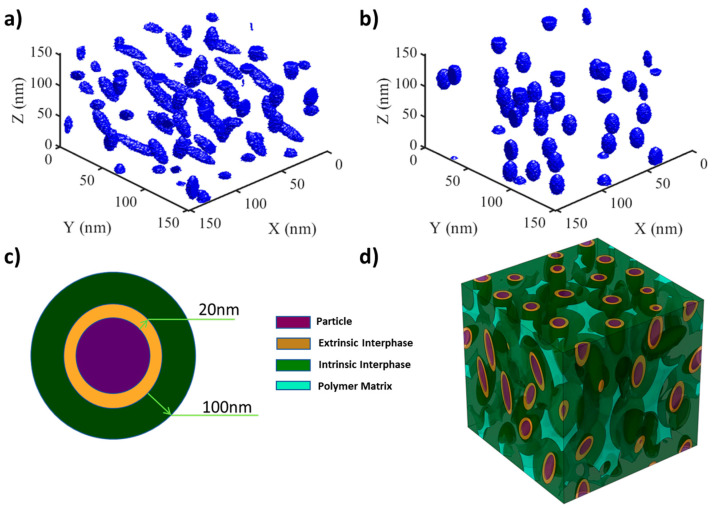
Nanodielectrics microstructure with varying characteristics (**a**,**b**) and polymer matrix and intrinsic interface properties (**c**,**d**). (**a**) High Aspect Ratio (AR), high Orientation Variation (OV), high Volume Fraction (VF), and low dispersion; (**b**) Low Aspect Ratio (AR), low Orientation Variation (OV), low Volume Fraction (VF), and high dispersion (**c**) schematic of extrinsic and intrinsic interface layer arrangement (**d**) microstructure representative volume element (RVE) where volume fraction, orientation, and aspect ratio of fillers vary.

To reconstruct the material systems, we implemented a simulated annealing algorithm to generate three-dimensional microstructure images with specified design characteristics and periodic boundary conditions. Initially, fillers are generated with predefined VF, AR, and OV. Next, the simulated annealing algorithm optimizes the structure to satisfy two important criteria. First, the reconstructed fillers must satisfy the predefined dispersion value, which is defined as the mean nearest neighbor between fillers. Second, the fillers must not collide to avoid violating a real physical system. These criteria are satisfied by checking the algebraic separation condition between each filler defined in [38]. Once both criteria are satisfied, the generated design is accepted for further analysis. Reconstruction is conducted based on a microstructure with the dimensions of [150 nm, 150 nm, 150 nm]. Figure 2a,b shows examples of reconstructions with varying characteristics.

##### Interfacial Layers

The material system studied here is TiO_2_ particles in a Cross-linked polyethylene (XLPE) polymer matrix. We simulate a multiphase system that considers, in addition to particle and matrix, extrinsic, and intrinsic interfacial regions (Figure 2c,d).

##### Extrinsic Interface

**Figure 3:**
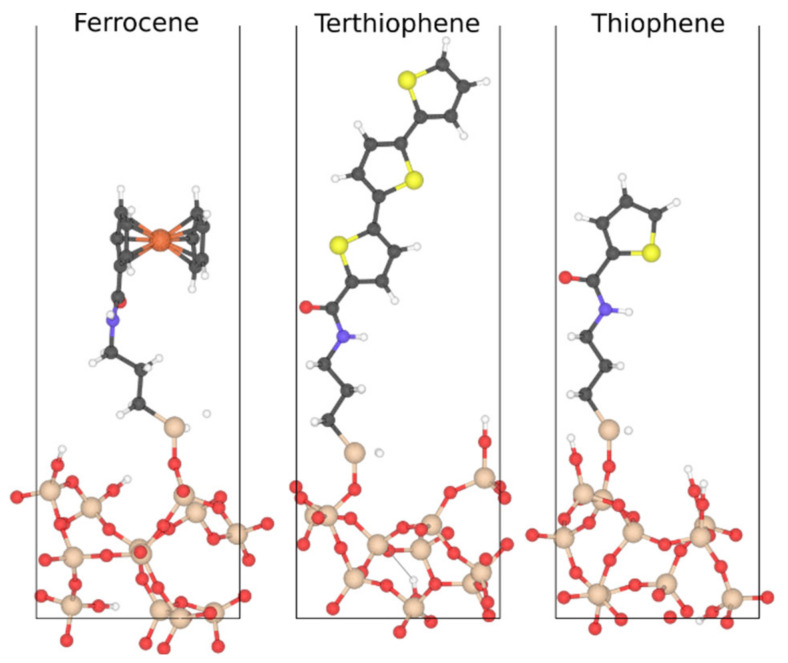
Characteristic structures of the three molecules considered here, attached to an amorphous surface, surrounded by polymer (not shown for clarity). We simulate an ensemble of 15 structures for each molecule to account for structural variations in the amorphous interface and predict trap distributions for estimating dielectric breakdown strength (Section First-Principles Predictions of Trap States).

The extrinsic interface simulates the effect of short chains grafted to the particles to enhance carrier trapping. We use three short ligand functionalization on the particle surface, as listed in Table 2 and with the molecular structure shown in Figure 3, to modify trap characteristics at the filler–matrix interface. For permittivity and loss calculations this extrinsic interface layer is modeled as a thin layer with conductivity based on a frequency-dependent loss characteristic: $\frac{-\sigma }{\omega \varepsilon_{0}}$. We have a fourth choice of ‘no extrinsic interface’ for which default interface trap configuration is used for breakdown strength calculation whereas no extrinsic phase is modelled for the permittivity-loss calculation.

##### Intrinsic Interface

The intrinsic interface is a result of particle–polymer interaction, with or without particle surface functionalization. This interaction can change the permittivity and loss (e.g., by reducing chain mobility, the polarizability is reduced decreasing permittivity, and changing the loss frequency). We model all possible qualitative combinations of changes in mobility and polarizability to capture a complete range of behaviors. This is conducted with the help of the Debye series framework combined with scale factors $S_{\alpha }$, $S_{\beta }$ for relaxation times $\tau_{n}$ and $M_{\alpha }$, $M_{\beta }$ for Debye coefficients $\Delta \varepsilon_{n}$. C is an offset to instantaneous permittivity component $\varepsilon_{\infty }$. Debye series, as shown in Equation (2), is a linear combination of complex basis functions with Debye coefficients $\Delta \varepsilon_{n}$ that act as weights.

(2) $$\varepsilon^{\prime }\left(\omega \right)=\varepsilon_{\infty }+\overset{N}{\underset{n=1}{\sum }}\frac{\Delta \varepsilon_{n}}{1+{\left(\omega \tau_{n}\right)}^{2}}\;\varepsilon^{\prime \prime }\left(\omega \right)=\overset{N}{\underset{n=1}{\sum }}\frac{\Delta \varepsilon_{n}\omega \tau_{n}}{1+{\left(\omega \tau_{n}\right)}^{2}}$$

The real part describes permittivity and the imaginary part describes loss characteristics. Table 3 shows these scale factors for all combinations considered. Debye series for the intrinsic interface, using a different set of scaling factors for α and β regime, is given by

(3) $$\varepsilon^{\prime }\left(\omega \right)=\varepsilon_{\infty }+C+M_{\alpha }\underset{\tau_{n}>\tau_{0}}{\sum }\frac{\Delta \varepsilon_{n}}{1+{\left(\omega S_{\alpha }\tau_{n}\right)}^{2}}+M_{\beta }\underset{\tau_{n}<\tau_{0}}{\sum }\frac{\Delta \varepsilon_{n}}{1+{\left(\omega S_{\beta }\tau_{n}\right)}^{2}}\;\varepsilon^{\prime \prime }\left(\omega \right)=M_{\alpha }\underset{\tau_{n}>\tau_{0}}{\sum }\frac{\Delta \varepsilon_{n}\omega \tau_{n}}{1+{\left(\omega S_{\alpha }\tau_{n}\right)}^{2}}+M_{\beta }\underset{\tau_{n}<\tau_{0}}{\sum }\frac{\Delta \varepsilon_{n}\omega \tau_{n}}{1+{\left(\omega S_{\beta }\tau_{n}\right)}^{2}}$$

$S_{\alpha },\;S_{\beta }<1$ implies repulsive interfacial interaction leading to faster relaxation of polarization entities moving overall relaxation spectra and loss peak to the right on the frequency axes, whereas $S_{\alpha },\;S_{\beta }>1$ implies the opposite behavior. $M_{\alpha }$ and $M_{\beta }$ modulate the magnitude of permittivity and loss contributed by each Debye element. We choose C to be zero assuming that the contribution from high frequency polarization mechanisms is not being altered due to molecular mobility changes brought on by interfacial interactions. Using separate scale factors enables control of alpha and beta regimes independently [20]. Although, for this design study, we assume that both the regimes exhibit changes in mobility and polarizability in the same qualitative way.

**Figure 4:**
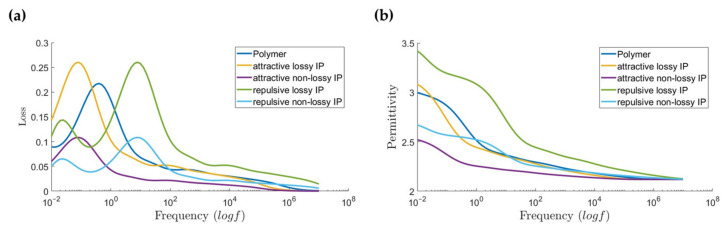
Polymer matrix and intrinsic interface properties (IP) corresponding to levels in Table 1 (**a**) Loss (**b**) Permittivity. *f* denotes frequency in Hz.

XLPE dielectric permittivity and loss data is taken from dielectric spectroscopy measurements and fitted to Equation (1) using a least square ridge regression with non-negative weights constraints. Figure 4 shows the XLPE matrix and interface material properties used for the simulations.

#### 2.2.3. Property Evaluation: Physics-Based Simulation Methods

##### Breakdown Strength Calculations

We perform Monte Carlo simulations of carrier hopping in an electronic energy landscape of the polymer nanocomposite to predict the mobility of carriers, following the procedure established and described in detail in [34]. We then calculate breakdown strength from the predicted mobility using a calibration of computed mobilities and experimentally measured breakdown strengths for microstructures extracted from the experiment, exactly as in [34].

The only modification to this overall process for the present work is in the energy landscape used for the Monte Carlo simulation. We previously accounted for the difference in electronic energy levels in the filler and polymer matrix to introduce trap states at the location of the fillers that control the carrier mobility, and hence, the breakdown strength. In the present study, we have different surface functionalizations that will each introduce different trap states in the extrinsic interface, in addition to those accounted for in the filler and matrix (including intrinsic interface) as before. We predict these trap states from the first principles as discussed below and incorporate them into the previously established simulation framework [34].

##### First-Principles Predictions of Trap States

Trap states critically impact electronic transport in polymers and nanocomposites but can only be indirectly inferred from experimental measurements such as transient depolarization current (TSDC) and luminescence spectroscopy, making it difficult to account for their influence on breakdown strength in material design. We previously developed a framework for systematic first-principles predictions of trap states at filler–polymer interfaces and extracted the multi-modal distribution of trap states at polyethylene–silica interfaces expected to significantly influence the carrier transport [39].

Here, we extend that approach to predict trap states at the extrinsic interfaces of functionalized fillers, focusing on molecules thiophene, terthiophene, and ferrocene at the inorganic–polymer interface (Figure 3). We create an ensemble of 15 amorphous interfaces containing each molecule, starting from random-walk polymer structures and classical molecular dynamics quench simulations followed by first-principles structure optimization to account for the randomness in the interfacial structure [39]. We then perform electronic density-functional theory calculations using the JDFTx plane-wave basis code [40], PBE-GGE exchange-correlation functional [41] with DFT-D2 dispersion corrections, GBRV ultrasoft pseudopotentials [42], and a kinetic energy cutoff of 20 and 100 Hartrees on the wavefunction and charge density, respectively, for each of these 45 large interfacial structures, each with approximately 300 atoms and 1000 valence electrons.

**Figure 5:**
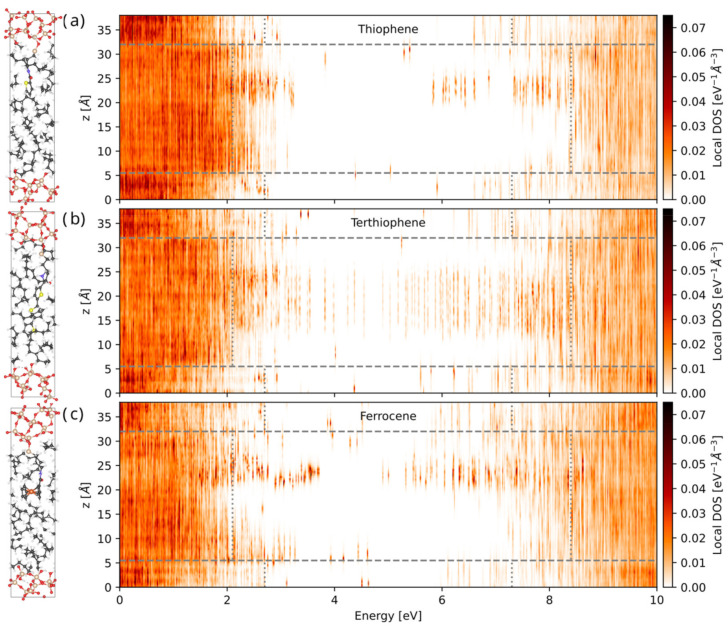
Ensemble-averaged local density of states (LDOS) of interfaces functionalized with (**a**) thiophene, (**b**) terthiophene, and (**c**) ferrocene, showing the energy and spatial distribution of trap states within the energy gap of these interfaces.

From these ensembles of electronic DFT calculations, we extract the ensemble-averaged, spatially resolved local density of electronic states (LDOS). Figure 5 shows the resulting LDOS for each of the three functionalized interfaces, with several localized energy levels visible at the interface within the energy gap of both the polymer and filler, which can act as trap states to reduce the carrier mobility and increase the breakdown strength of the nanocomposite. The thiophene-functionalized interface exhibits relatively fewer trap states that are located closer to the conduction band, while the terthiophene case exhibits a greater number of traps states that are more evenly spread out in energy and in space due to the conjugated electronic structure of terthiophene. Ferrocene contains the most trap states, which are localized and deeper in energy (closer to the center of the band gap), indicating the most potential for carrier trapping and high breakdown strength.

**Figure 6:**
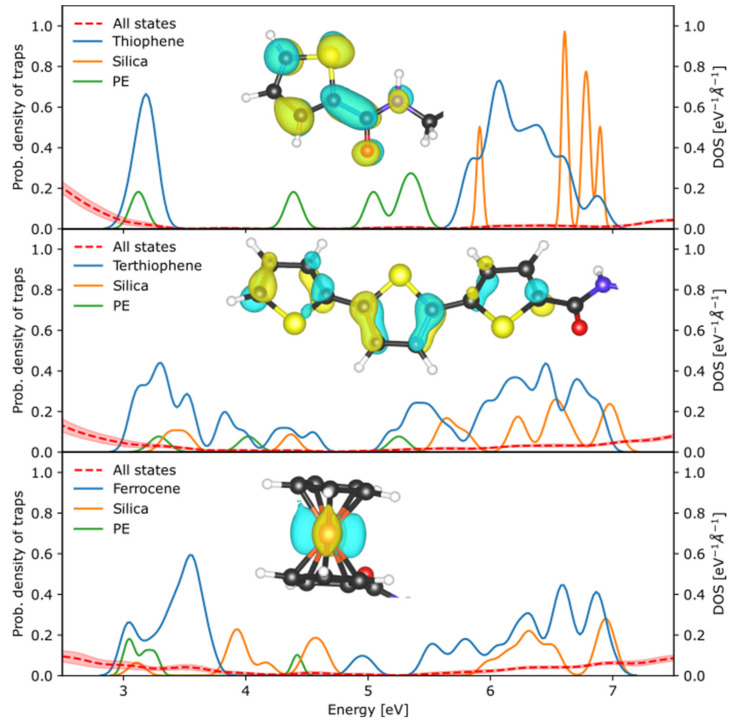
Probability distribution of trap states introduced by the three functional groups with inset visualizing the electronic orbital associated with one of the trap states on each molecule.

Finally, Figure 6 summarizes the energy distribution of these trap states extracted from Figure 5, along with the orbitals of a characteristic trap state on the molecule in the extrinsic interface. Thiophene and terthiophene trap states emerge from states delocalized across the conjugated bonds, with a greater spatial extent for terthiophene, while the dominant ferrocene trap states are localized *d* orbitals of the iron atom. We sample energies from the above distributions to populate the energy landscape of the extrinsic interface region of the Monte Carlo simulations, thereby combining these first-principles materials input with the microstructure of fillers within the matrix in the overall breakdown strength simulation. We expect ferrocene to exhibit the maximum reduction in carrier mobility and associated increase in breakdown strength, due to its large number of shallow and deep trap states covering a large energy range, followed by terthiophene and then thiophene.

##### Permittivity and Loss Calculations

We model permittivity and loss properties using a continuum scale model. Each microstructure is modelled as a multiphase continuum system consisting of four (4) phases namely, polymer matrix, intrinsic interface layer, extrinsic interface layer, and particles (Figure 2c,d). Details of methods to calculate continuum scale property values for extrinsic and intrinsic layers are explained in Section Interfacial layers. Further, these microstructure domains are discretized using a finite difference method to solve Poisson’s equation describing a complex-valued scalar potential field for dynamic (in frequency domain) steady-state currents. Effective permittivity and loss of the representative volume element (RVE)s is calculated using average electric displacement across the RVE walls in real and imaginary parts, respectively. Because of the anisotropy in the system, we do a full permittivity tensor calculation by changing the field application directions. This amounts to three (3) calculations per data point. The frequency range considered for these applications is $10^{-2}$ Hz to $10^{7}$ Hz. The main highlight of this method is our in-house implementation of the code that uses the Hermitian matrix to solve linear systems of equations in complex spatial potential. We employ structural and Dirichlet periodic boundary conditions for microstructure RVEs to effectively mimic a larger continuum. The authors would like to mention that COMSOL finite element simulations, while effective for their high-order field value accuracies and readily available visualization capabilities, were rather difficult to integrate into the BO optimization loop. The reasons involved auto geometry and auto mesh build failures for complex geometries with high VF, and high AR systems as well as high compute memory, build time and computational time requirement for 3D microstructures with number of elements exceeding ten million.

#### 2.2.4. Metamodeling and Multi-Objective Optimization

##### Latent Variable Gaussian Process (LVGP) for Metamodeling

Gaussian processes (GPs) are a specific interpolation-based machine learning model that has been a popular choice for many engineering applications due to their ability to provide both objective and uncertainty prediction. Although very powerful, one caveat with GP metamodels is that due to the nature of their correlation functions, GP models can only handle quantitative variables, and qualitative variables cannot be implemented directly without a well-defined distance metric. However, for all physical qualitative variables, there exists an underlying, potentially high-dimensional, quantitative representation that explains the qualitative variables’ impact on the response. Using this knowledge, a novel Latent Variable Gaussian Process (LVGP) metamodel that approximates the effects of underlying quantitative variables through an implicit mapping from qualitative variables into a low-dimensional quantitative latent has been developed [43]. With LVGP, qualitative variables, along with the quantitative variables can be used together to provide mixed-variable GP modeling. The spatial relationship of the latent variables provides interpretability regarding the relationship of qualitative variables with the response and the LVGP approach provides predictions and quantifies uncertainties, making it very suitable for Bayesian optimization applications. As a result, we implemented the LVGP machine learning model to learn the relationship between the mixed-variable nanodielectrics design parameters and properties. The mathematical formulation of LVGP is provided in Appendix A.

##### Bayesian Optimization

Our main goal in this study is to optimize the design of nanodielectric materials for the two conflicting capacitor properties in three directions (X, Y, Z). Explicitly, we would like to minimize the dielectric losses and maximize the stored energy density values in all three directions. Bayesian optimization (BO) is a well-known surrogate model-based optimization methodology that has been previously implemented for optimization problems. With its advantageous characteristics such as ease of implementation, and gradient-free optimization, it has been widely used in the materials design community [35,44,45,46]. For multi-objective optimization problems with a large number of objectives, a simple but promising approach is to incorporate all objectives into a single objective. In this formulation, each property is treated equally and optimized at the same time. For the design optimization of nanodielectric materials, the single objective multi-criteria function is defined as

(4) $$y\left(x\right)={\varepsilon^{\prime \prime }}_{r}^{x}+{\varepsilon^{\prime \prime }}_{r}^{y}+{\varepsilon^{\prime \prime }}_{r}^{z}-U_{d}^{x}-U_{d}^{y}-U_{d}^{z},$$

where $\in^{i}$, $U_{d}^{i}$ are the normalized dielectric loss and stored energy density properties for three directions (i=x,y,z). BO selects the candidate designs through maximizing an acquisition function called the Expected Improvement (*EI*) that aims to balance the exploration and exploitation of the design space. BO employs a surrogate model that can provide both predictions and uncertainty quantification to evaluate the acquisition function. Considering the mixed-variable design space, the LVGP is a very suitable choice of surrogate model. Therefore, the EI acquisition function can be formulated as

(5) $$EI\left(x\right)=\left(y^{*}-{y_{x}}^{\prime }\;\right)\cdot \psi \left(\frac{y^{*}-y_{x}^{\prime }}{\sigma_{x}}\;\right)+\sigma_{x}\cdot \phi \left(\frac{y^{*}-y_{x}^{\prime }}{\sigma_{x}}\;\right)\;,$$

where $y^{*}$ is the lowest objective value observed so far in the optimization, $y_{x}^{\prime }$ and $\sigma_{x}$ are the predicted objective and prediction uncertainty from LVGP at candidate design point x, ψ is the cumulative distribution function (CDF), and ϕ is the probability density function (PDF). The EI is calculated for all design candidates and the candidate with the highest EI value is selected for property evaluations in each BO iteration.

#### 2.2.5. Design Analysis

Once the optimization is completed, we analyze the results obtained from the framework to identify novel designs and make conclusions regarding the design of nanodielectric materials.

### 2.3. Global Sensitivity Analysis for the Mixed-Variable Design Space

Global Sensitivity analysis (GSA) is the study of how the input design variables, independently or interactively, influence the design objective. In the context of this nanodielectrics design study, GSA can help us understand the influence of the six design variables, individually or interactively, on the material properties. Among different types of GSA methods, variance-based methods, specifically Sobol’s sensitivity indices are well-known methods to explain the contribution of each design variable on the variability of the response. Specifically, the two Sobol’s indices, Main Sensitivity Index (MSI) and the Total Sensitivity Index (TSI), quantify how each design variable contributes to the response individually and interactively with other design variables [47]. For a given design space with q number of design variables, the formulation of Sobol’s indices, TSI and MSI are given in Equations (6) and (7).

(6) $$S_{i}=\frac{Var_{x}\left[E_{x_{1},\ldots ,x_{q}}y|x_{i}\right]}{Var_{x_{1},\ldots ,x_{q}}\;\left[y\right]}=\frac{V_{i}}{V},$$

(7) $$S_{i}^{t}=S_{i}+S_{i,\sim i},\;.$$

Here, *y* is the response of interest, $V_{i}$ is the variance of the response with respect to the changes in design variable $x_{i}$, *V* is the variance of the response, $S_{i}$ is the MSI, $S_{i}^{t}$ is the TSI of the design variables, and $S_{i,\sim i}$ is the higher order Sobol’ sensitivity indices between variable $x_{i}$ and remaining variables $x_{i}$ for i=1,…,q.

Current Sobol’ indices calculations require a large number of objective evaluations and are limited to quantitative design spaces only. With its capability of capturing the relationship between mixed-variable design spaces and providing fast and accurate response predictions, we can overcome the aforementioned challenges by employing LVGP as a metamodel to create a mixed-variable sensitivity analysis to incorporate qualitative variables into sensitivity studies [48]. To conduct the mixed-variable GSA, the mixed-variable samples are first fed into the LVGP model for model fitting. Once the trained model is ready, Sobol’ analysis is conducted through evaluating the Sobol’ sensitivity metrics, Main Sensitivity Index (MSI) and Total Sensitivity Index (TSI), given in Equations (6) and (7) using the fast and accurate predictions provided by the LVGP model.

## 3. Results

Here, we present in Section 3.1, findings from the Base DoE of 100 nanodielectric material designs that were simulated using the multiscale physics-based method described in Section 2. Permittivity and loss simulations were performed in a full frequency space whereas breakdown strength calculation was conducted in the limit of zero frequency. Properties were calculated by applying the external field in three spatial directions (X, Y, Z) due to the anisotropy of many of the samples in the DOE, where the default orientation of particles is always in the Z direction. GSA with Sobol’s analysis performed on these computational results was used to highlight the sensitivity of property values. Section 3.2 presents the results obtained from design optimization with ML model fitting and Bayesian optimization at frequencies 60 Hz and 1000 Hz, two potential frequency regimes for capacitive applications.

### 3.1. Initial Design of Experiments (DOE)

For the initial DOE points, we looked at the simulated property data scatter and linear correlation between three basic dielectric properties—permittivity, loss, and breakdown strength at the two representative frequencies 1000 Hz and 60 Hz. We also calculated the linear correlation between stored energy density (SED) and loss as that pairing is more relevant for applications and is used further to perform design optimization. We observe a strong correlation between permittivity and loss (Table 4) which reflects the expected strong fundamental mechanistic coupling between those entities. On the other hand, an overall weak correlation between breakdown strength and loss was observed. This result underscores that the mechanism of breakdown strength modeled here is not directly coupled with polarization processes. Furthermore, combining breakdown strength and permittivity in Equation (1) produces a moderate correlation between stored energy density and loss, quantities that we put together in the Bayesian optimization.

**Figure 7:**
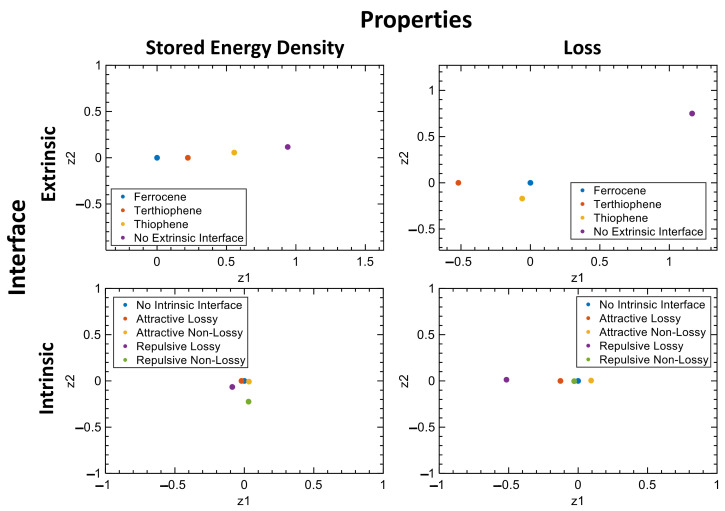
The latent variables of the extrinsic and intrinsic interface design choices were obtained from the LVGP models trained on the initial DOE at 60 Hz in the Z direction. Here, the axis (z1, z2) represents the latent variables obtained from the LVGP model.

Next, we fit our machine learning model, LVGP, to learn the relationships between the design variables and the two dielectric properties that we are interested in optimizing, SED and Loss. Figure 7 shows the latent variables obtained for the two properties at 60 Hz. Here, each point corresponds to the latent variable of the interface design choices. For the SED, we observe that the latent variables of qualitative (categorial) extrinsic interface choices are further apart from each other, suggesting that their influence on the response are much different from each other. In contrast, the intrinsic interface latent variables are much closer in the latent space, suggesting that the choice of interface has a similar influence on the SED. The ferrocene and thiophene choices have a similar influence on Loss, whereas using no extrinsic interface or terthiophene could have a significant influence on the Loss. Finally, although not shown here, similar observations were made for the latent variables obtained at 1000 Hz.

**Figure 8:**
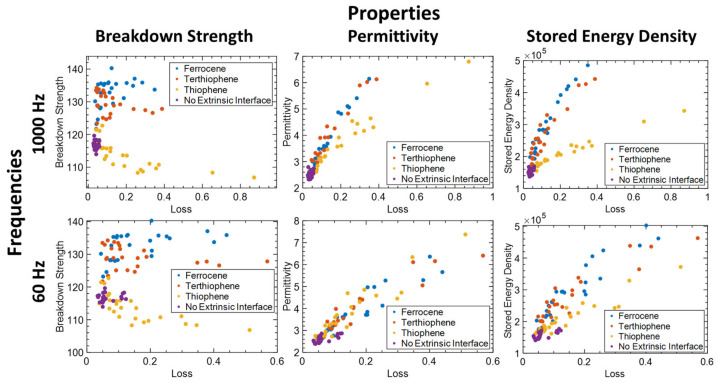
Nanodielectrics properties in the z direction based on the DOE designs at two frequencies 1000 Hz and 60 Hz with respect to extrinsic interface design choices.

To investigate and understand the influence of interface further, we looked at the scatter plots at 60 Hz and 1000 Hz for properties in the z direction, with color coding of data points according to extrinsic interface choice (Figure 8). First, we observed that the addition of an extrinsic interface of charge-trapping molecules significantly improves breakdown strength and permittivity over cases without any extrinsic interface (purple vs other colors). However, this results in a significant increase in loss. Ferrocene results in the highest breakdown strength which we attribute to the deeper trap distribution as determined from DFT (blue vs. other colors). This finding is consistent with other work [27].

At 1000 Hz, composites with a thiophene extrinsic interface exhibit lower permittivity compared to composites with terthiophene and ferrocene, whereas the permittivity is comparable at 60 Hz (yellow vs other colors). Additionally, the thiophene results in the largest loss at 1000 Hz. Finally, because of the above, the stored energy density for thiophene at 1000 Hz is significantly lower than ferrocene and terthiophene; however, it improves at 60 Hz owing to improved permittivity at that frequency. For completeness of data presentation, scatter plots and correlation indices for the X and Y direction are included in Supporting Information.

#### Global Sensitivity Analysis

**Figure 9:**
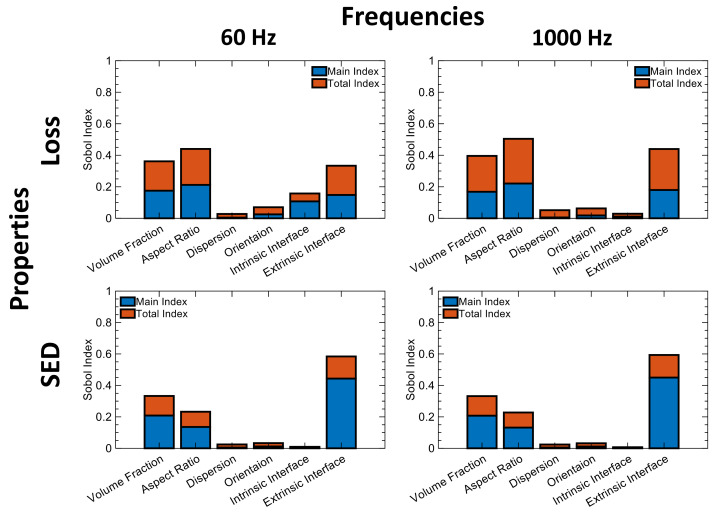
GSA of two Z components of design objectives, namely stored energy density and loss with respect to design variables, both quantitative and qualitative.

The GSA is used to gain knowledge of the cause–effect relationship and further validate whether the simulation model created in (Section 2.2.4) matches the underlying physical behavior. To extract the influence of design choices on the properties, we have performed LVGP-based mixed-variable global sensitivity analysis (GSA) on the initial DOE designs. The GSA aims to explain how each design variable contributes to the variability of the properties both individually and collectively. To conduct GSA, we fitted LVGP models on SED and Loss in the Z direction at 60 Hz and 1000 Hz frequencies. Figure 9 shows Sobol’s sensitivity indices obtained from the GSA for each design variable. In the figure, blue bars describe the individual contribution of each design variable on the response (Main Index) and orange bars describe the interactive contribution of the design variable with other variables on the response (Total Index).

**Figure 10:**
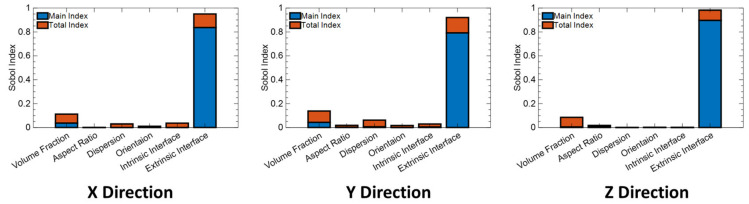
GSA of breakdown strength with respect to design variables, both quantitative and qualitative.

We observe that the SED is highly sensitive to the choice of the extrinsic interface on its own, consistent with the prior latent variable analysis in Figure 7 and demonstrating its power to extract physical insight. There is also moderate interaction between extrinsic interface choice, particle VF and AR to influence variability in SED which means a particular combination of these three design variables can make a big difference in SED. Similarly, for loss, we observe that the choice of the interface along with the aspect ratio and volume fraction contribute to a significant variance in the loss. Furthermore, we observe high Total Sensitivity Index (TSI) values between those design variables, meaning that they interactively influence the loss. We also note that the choice of the intrinsic interface can play a role in the variability of loss at lower frequencies as the latent variables suggested before (Figure 8). We further investigated the individual GSA of permittivity and breakdown strength. Breakdown strength is highly sensitive to extrinsic interface and to some extent to particle VF (Figure 10). A similar GSA for permittivity is shown in the Supporting Information (Figure S2), demonstrating the influence of the microstructural parameters and their frequency dependence.

These results on the significant influence and interaction of these parameters partially confirm our hypothesis of the existence of a microstructural parameter space that can result in improved SED while mitigating increases in loss. Combinations of VF, AR, and extrinsic layers are consistently influential at low and high frequencies, whereas orientation, intrinsic interface, and particle dispersion play a smaller role that varies with frequency.

### 3.2. Nanodielectrics Design Optimization

**Figure 11:**
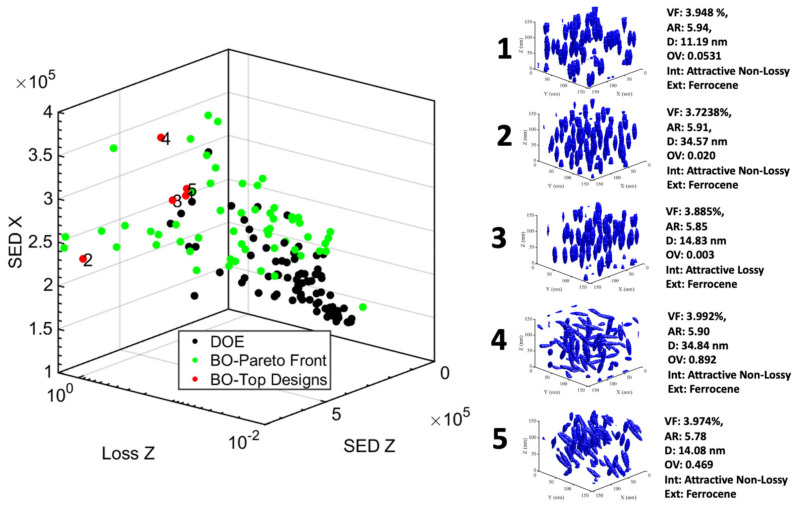
The Loss (Z direction) and Stored Energy Density (Z and X direction) properties of 100 DOE designs (black) against the Top 5 (red) and Pareto front (light green) designs were obtained after design optimization at 60 Hz.

**Figure 12:**
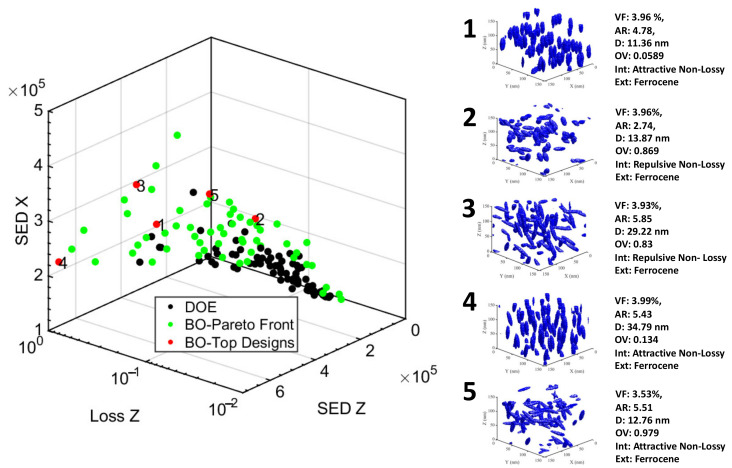
The Loss (Z direction) and Stored Energy Density (Z and X direction) properties of 100 DOE designs (black) against the Top 5 (red) and Pareto front (light green) designs were obtained after design optimization at 1000 Hz.

Armed with the promising sensitivity analysis, we built our LVGP machine learning model based on the 100 sample designs from the base DOE for two frequencies (60 Hz and 1000 Hz) and initiated Bayesian optimization to identify the nanodielectric designs that optimize the single-objective multi-criteria defined in Section Bayesian Optimization. Figure 11 and Figure 12 show the top five (5) designs (red) obtained from BO in a sub-space of three out of six optimized properties against the base DOE (black) and the indirectly optimized Pareto front (light green) designs from BO for the operating frequencies 60 Hz and 1000 Hz, respectively. Additionally, we have visualized the microstructure of each of those top five designs and listed their design variable values.

We observed that these designs are dominated by high VF (maximum of the range allowed is 4), high AR (maximum of the range is 6), ferrocene as an extrinsic interface, and a non-lossy intrinsic interface layer. Furthermore, we see a comparable emphasis between isotropic and anisotropic structures (OV) as well as low and high dispersion (D). The reason behind high VF design choices is that the levels of volume fractions used in these systems are still well below particle percolation network threshold that can lead to connected network between particles leading to any leakage currents and resulting losses [1,49].

**Figure 13:**
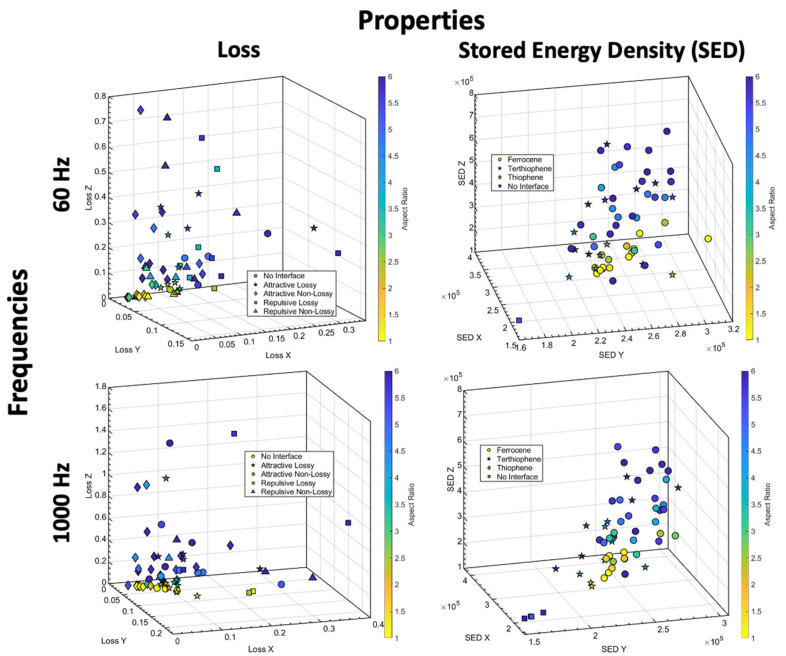
Pareto front designs are plotted in loss space and SED space. A color bar is used to denote the Aspect Ratio (AR) of the Pareto front designs and shapes are used to denote the intrinsic layer choices on the loss plot (**left column**) whereas shapes denote extrinsic layer choices in SED plot (**right column**).

To understand what is driving the choice of AR, extrinsic and intrinsic values, we looked at the entire Pareto front. Figure 13 shows all Pareto points designs in SED components space and loss components space, with coloring to denote AR. We see that the low-loss corner of this space is predominantly populated by combinations of moderate AR (especially around low loss in the Z direction) with ANL and occasionally with RNL. On the other hand, in SED space, the high values corner is packed with high AR combined with ferrocene and few with thiophene extrinsic interfaces. Thus, we infer that the choice of the intrinsic layer is driven by loss minimization whereas the choice of AR and extrinsic layer is driven by SED maximization.

Next, to analyze choices of OV and dispersion, we looked at the SED and loss in all three directions for the top designs from Figure 11 and Figure 12 listed in Table 5 and Table 6. It is apparent that to maximize total SED, the model either tries to increase SED in one direction (here, Z-direction) significantly or to combine moderate increase in SED in all directions. Combination of high alignment (anisotropy, low OV)) and high dispersion (D) results in high SED in the direction of alignment (z-direction), as seen in design 2 at 60 Hz and design 4 at 1000 Hz. On the other hand, combinations of isotropy (high OV) and high dispersion produces a moderate balance of SED in all three directions, as witnessed in design 4 for 60 Hz and design 3 for 1000 Hz.

It is determined that the above changes in SED values across shown designs can be attributed to changes in permittivity as the extrinsic interface choice is identical across all the designs. Extrinsic interface choice predominantly drives changes in breakdown strength as seen in the sensitivity analysis of Section 3.1, Figure 10. The enhancement in permittivity with higher particle alignment is attributed to the interaction between field concentrations around individual particles which reduces as the particles cluster (e.g., design 1 vs. 4 at 1000 Hz). Additionally, if the particles are dispersed isotropically (high OV), the properties are isotropic. Relative increases or decreases in loss are interlinked with these changes in permittivity due to mechanistic coupling between permittivity and loss.

Lastly, we observe that lower losses among these designs are attained by either reducing the above-explained interactions through lower dispersion (design 1 at 60 Hz) or diffusing those interactions in other directions through isotropy (design 5 at 1000 Hz). Design 2 at 1000 Hz has relatively lower losses in all three directions. However, it could do so by choosing smaller AR and at the cost of reduced total stored energy density (through reduced permittivity) compared to other designs.

## 4. Discussion

We hypothesized that there exists a parameter space that combines moderately high nanoparticle aspect ratios, extrinsic carrier trapping functionalization, and intrinsic interface characteristics that can enable increasing breakdown strength and dielectric constant simultaneously and minimize increases in loss. For this, we simulated a high dimensional mixed variable base DoE of size 100 using multiscale physics-based property computation. Using this base DoE, we cast the design of polymer nanodielectrics for capacitive energy storage as a mixed-variable, multi-objective design problem and identify the optimized designs between two fundamental capacitor device properties: Dielectric Loss and Stored Energy Density. We integrated a Latent Variable Gaussian Process machine learning model along with Bayesian optimization to approach the design challenge at hand. Finally, we implemented a mixed-variable metamodel-based global sensitivity analysis to gain and extract physical insights into the collective relation between design variables and output properties of base DoE. We further used this insight to understand and assess optimized design choices that emerged in the process.

The use of physics-based computational and machine learning-based optimization algorithms helps explore this design space at a much quicker pace compared to experimental design. Our design framework was able to identify promising dielectric properties that contribute to enlarging property space between two conflicting properties. We found through the design choices made by our metamodel, that a modest aspect ratio combined with deeper surface trap densities and a non-lossy intrinsic interface layer can achieve simultaneous increases in nanocomposite breakdown strength and dielectric constant while limiting increases in loss. Here, among the extrinsic layer choices, ferrocene, with deeper traps, shows the highest improvement in breakdown strength. Increases in simulated effective nanocomposite permittivity is due to particle aspect ratio and additionally due to interfacial polarization introduced by charge activity (modelled as conductivity-based loss component) of extrinsic layer. However, both strategies increase loss substantially. Our optimal solutions tried to keep losses low by choosing a non-lossy intrinsic interface. The design process also demonstrated that the interplay between particle alignment and dispersion can yield optimal solutions with any combination of extreme values of each descriptor through field concentration interactions. As seen in the past literature [9,50], losses go up quickly as one employs means of increasing permittivity; however, some of our design choices were able to keep the losses around 0.1. In our opinion, this is a key design variable that has significant potential for further development by designing an intrinsic layer to drive losses down even further.

Furthermore, we conclude that the simulated property trends are consistent with the collective understanding of polymer nanodielectrics systems based upon past experimental observations and multiscale physics such as polarization phenomena and electron carrier hopping. The learned surrogate model predictions match well with the explicit DOE simulations which are rooted in the physics of dielectric processes. A well-formed Pareto front advanced towards the high stored energy density and low loss with efficient sampling iterations. These observations verify the quality of the metamodel. The GSA finding indicated interactions within the design variables and helped consolidate the hypothesis of the existence of niche design space and validated our analysis of the interaction between dispersion and degree of particle alignment.

However, this discussion would be incomplete without acknowledging some of the limitations of the simulation methods. In the permittivity and loss model, the properties of each interface layer are modelled as averages; specifically, the assumed conductivity of the extrinsic layer is a bulk-level property value. Our model captures interfacial polarization induced by charge transport via (1) the conductivity-based loss component ($\in^{\prime \prime }$) for particles and the extrinsic interface and (2) the mobility characteristics for the intrinsic interfacial layer and the polymer which are modulated by frequency. Previous studies that have tried to analyze and provide insight into interfacial polarization underline the importance of local mobility [14,51]. It is important to differentiate between backbone mobility and polar side group mobility [17,52,53]. For modelling effects of interfacial polarization via charge transport simulation more accurately, it will be necessary to go beyond current dielectric spectrum measurements which are averaged over the bulk. The same is true for modelling charge transport through the intrinsic layer and is equally crucial as the intrinsic layer is a significant part of the total phase composition. While the simulation results can be improved, the data analysis and design approach presented in this work are generally applicable to a wide range of nanocomposite and other materials designs where categorical and continuous design variables co-exist.

## Figures and Tables

**Table 1:** The Mixed Variable Nanodielectrics Design Space with bounds on the variable ranges. The dispersion parameter refers to the nearest neighbor distance.

	Design Variables	Design Choices
** Microstructural (Quantitative) **	Volume Fraction (VF)	(1,4)%
	Aspect Ratio (AR)	(1–6)
	Dispersion (D)	(11–36) nm
	Orientation Variation (OV)	(0,1)
** Interfacial (Qualitative) **	Intrinsic Interface	Attractive Lossy, Attractive Non-Lossy, Repulsive Lossy, Repulsive Non-Lossy, No Interface
	Extrinsic Interface	Ferrocene, Terthiophene, Thiophene, No Interface (No Extrinsic Interface)

**Table 2:** List of the molecule choices for the extrinsic interface and the conductivity used in the modeling.

#	Ligand Molecule	Conductivity $\sigma \;\left(S/\mathrm{cm}\right)$
1	Thiophene	1 × 10^−10^
2	Terthiophene	1 × 10^−7^
3	Ferrocene	1 × 10^−1^

**Table 3:** List of the intrinsic interface choices and the associated parameters used in the modeling.

#	Intrinsic Interface	$S_{\beta }$	$M_{\beta }$	$S_{\alpha }$	$M_{\alpha }$	C
1	Attractive Lossy (AL)	5.0	1.2	7.0	1.1	0
2	Attractive Non-Lossy (ANL)	5.0	0.5	7.0	0.5	0
3	Repulsive Lossy (RL)	0.05	1.2	0.07	1.1	0
4	Repulsive Non-Lossy (RNL)	0.05	0.5	0.07	0.5	0
5	No Intrinsic Interface	1	1	1	1	0

**Table 4:** Linear correlation between dielectric properties in different directions at 60 Hz.

Properties/Directions	X	Y	Z
Loss vs. Breakdown Strength	0.21	0.2	0.12
Loss vs. Permittivity	0.78	0.7	0.93
Loss vs. Stored Energy Density	0.63	0.54	0.85

**Table 5:** Loss and SED components in x, y, and z directions for top 5 designs (visualized in Figure 11) at 60 Hz.

Top Designs/Properties	Loss_x	Loss_y	Loss_z	$\mathrm{SED}\_x\;\left(\times 10^{2}\right)$	$\mathrm{SED}\_y\;\left(\times 10^{2}\right)$	$\mathrm{SED}\_z\;\left(\times 10^{2}\right)$
1	0.068	0.041	0.309	2976	2411	5060
2	0.038	0.035	0.943	2364	2325	7709
3	0.108	0.074	0.381	2921	2709	5276
4	0.194	0.090	0.460	3638	2854	5459
5	0.117	0.104	0.319	3043	2941	4952

**Table 6:** Loss and SED components in x, y and z directions for top 5 (visualized in Figure 12) at 1000 Hz.

Top Designs/Properties	Loss_x	Loss_y	Loss_z	$\mathrm{SED}\_x\;\left(\times 10^{2}\right)$	$\mathrm{SED}\_y\;\left(\times 10^{2}\right)$	$\mathrm{SED}\_z\;\left(\times 10^{2}\right)$
1	0.055	0.033	0.300	2759	2432	4679
2	0.071	0.062	0.073	2880	2882	2931
3	0.179	0.074	0.376	3512	2885	5150
4	0.030	0.032	0.774	2316	2341	7238
5	0.107	0.077	0.153	3272	2828	3590

## Data Availability

Data for microstructures and properties are being made available at materialsmine.org.

## Supplementary Materials

The following supporting information can be downloaded at: https://www.mdpi.com/article/10.3390/nano13172394/s1, Table S1. List of abbreviations used in the manuscript. Figure S1. Nanodielectrics properties in the z direction based on the DOE designs at two frequencies 1000 Hz and 60 Hz with respect to intrinsic interface design choices. Figure S2. Global Sensitivity Analysis on the difference in permittivity at 1 × 10^−2^ Hz and 1 × 10^7^ Hz with respect to the mixed-variable design space.

## Appendix A. Latent Variable Gaussian Process (LVGP) Modeling

Given a mixed-variable design space with $w={\left[x^{T},t^{T}\right]}^{T}$, where $x={\left[x_{1},x_{2},\ldots ,x_{q}\;\right]}^{T}\in R^{q}$ are quantitative design variables and $t={\left[t_{1},t_{2},\ldots ,t_{m}\right]}^{T}$ are qualitative design variables. Each qualitative design variable $t_{j}$ has $l_{j}$ design options (levels), i.e., $t_{j}\in \left\{1,2,\ldots ,l_{j}\right\}$ for j=1,2,…m. For real physical models, there are quantitative variables $v\left(t_{j}\right)=\left[v_{1}\left(t_{j}\right),v_{2}\left(t_{j}\right)\ldots ,v_{n}\left(t_{j}\right)\right]\in R^{n}$ underlying each qualitative variable, which could be extremely high-dimensional. The key idea of LVGP is to learn a low-dimensional latent space to approximate the original underlying qualitative space via statistical inference. Although the dimensions of the latent variable vector $z\;\in \;R^{k}$ can be freely chosen, a two-dimensional (2D) latent vector, k=2, is usually sufficient in most engineering designs, which is also adopted in this study. Thus, each level of a qualitative variable $t_{j}$ is represented by a 2D latent vector $z\left(t_{j}\right)={\left[z_{j,1}\;,z_{j,2}\;\right]}^{T}$. The transformed design space becomes $h=\left[x^{T},z{\left(t\right)}^{T}\right]\in R^{\left(q+m\times 2\right)}$, where $z\left(t\right)={\left[z_{1,1},z_{1,2},\ldots ,z_{j,1},z_{j,2},\ldots ,z_{m,1},z_{m,2}\right]}^{T}$.

Now, consider a single response GP model with a prior constant mean μ to describe the mean response at any given point in the design space h. A zero-mean Gaussian Process is used to capture the variance of the response, described by a covariance function $K\left(h,h^{\prime }\right)$. The covariance function $K\left(h,h^{\prime }\right)=\sigma^{2}\cdot c\left(h,h^{\prime }\right)$ describes the relationship or the correlation of responses at any pairs of input points h and $h^{\prime }$, where $\sigma^{2}$ represents the prior variance of the GP model and $c\left(h,h^{\prime }\right)$ is the correlation function. LVGP extends the commonly used Gaussian correlation function to include latent variables,

$$c\left(h,h^{\prime }\right)=\exp\left(\overset{q}{\underset{i=1}{\sum }}\phi_{i}{\left(x_{i}-x_{i}^{\prime }\right)}^{2}-\overset{m}{\underset{j=1}{\sum }}\|z_{j,1}-z_{j,1}^{\prime }{\|}_{2}^{2}+\|z_{j,2}-z_{j,2}^{\prime }{\|}_{2}^{2}\;\right)$$

where $\phi_{i}$ is a scaling parameter that will be estimated for each quantitative variable $x_{i}$. The mapping from qualitative variables to 2D latent variables is scaled so that the scaling parameters of latent variables $z\left(t\right)$ is a unity vector, are set to be 1 as they will be estimated as hyperparameters. The rationale behind this correlation function is that points closed in the design space h should also exhibit similar output patterns. For a given design space with n number of points, the parameters, μ, σ, and ϕ, along with the 2D mapped latent variables, $z\left(t\right)$, are estimated through Maximum Likelihood Estimation (MLE), i.e., finding parameters to maximize the log-likelihood function,

$$l\left(\mu ,\sigma ,\phi ,z\right)=-\frac{n}{2}\ln\left(\sigma^{2}\right)-\frac{1}{2}\ln\left|c\left(z,\;\phi \right)\right|-\frac{1}{2\sigma^{2}\;}\;{\left(y-\mu 1\right)}^{T}c{\left(z,\phi \right)}^{-1}\;\left(y-\mu 1\right),$$

where C is the n×n correlation matrix with $C_{ij}=c\left(h^{i},h^{j}\right)$ for i,j=1,2,3,…,n, 1 is a vector of ones with dimensions of n×1, and $y={\left[y_{1},y_{2},\ldots ,y_{n}\right]}^{T}$ is the observed response vector. Readers interested further in LVGP are referred to the original paper [43].
