# Supplementary material for: Design of Polymer Nanodielectrics for Capacitive Energy Storage
Source: Nanomaterials (Basel). 2023 Aug 22;13(17):2394. doi: 10.3390/nano13172394 (PMC10490420; doi:10.3390/nano13172394)
Supplement: Supplementary file 1 [file nanomaterials-13-02394-s001.zip › nanomaterials-2497269-supplementary.pdf]

nanomaterials

Supplementary Materials

Design of Polymer Nanodielectrics for Capacitive Energy Storage

Prajakta Prabhune [1,†], Yigitcan Comlek [2,†], Abhishek Shandilya [3], Ravishankar Sundararaman [3], Linda S. Schadler [4], Lynda Catherine Brinson [1] and Wei Chen [2,*]

1 Thomas Lord Department of Mechanical Engineering and Material Science, Duke University, Durham, NC 27708, USA; prajakta.prabhune@duke.edu (P.P.); cate.brinson@duke.edu (L.C.B.)
2 Department of Mechanical Engineering, Northwestern University, Evanston, IL 60208, USA; yigitcancomlek2024@u.northwestern.edu
3 Materials Science and Engineering, Rensselaer Polytechnic Institute, Troy, NY 12180, USA; abhishandy@protonmail.com (A.S.); sundar@rpi.edu (R.S.)
4 College of Engineering and Mathematical Sciences, University of Vermont, Burlington, VT 05405, USA; linda.schadler@uvm.edu
* Correspondence: weichen@northwestern.edu
† These authors contributed equally to this work.

Table S1. List of abbreviations used in the manuscript.

Abbreviations	Explanation
SED	Stored Energy Density
GP	Gaussian Process
LVGP	Latent Variable Gaussian Process
BO	Bayesian Optimization
XLPE	Cross-linked Polyethylene
VF	Volume Fraction
AR	Aspect Ration
OV	Orientation Variation
D	Dispersion
DOE	Design of Experiments
GSA	Global Sensitivity Analysis
MSI	Main Sensitivity Index
TSI	Total Sensitivity Index
AL	Attractive Lossy
ANL	Attractive Non-Lossy
RL	Repulsive Lossy
RNL	Repulsive Non-Lossy
LDOS	Local Density of Electronic States
RVE	Representative Volume Element

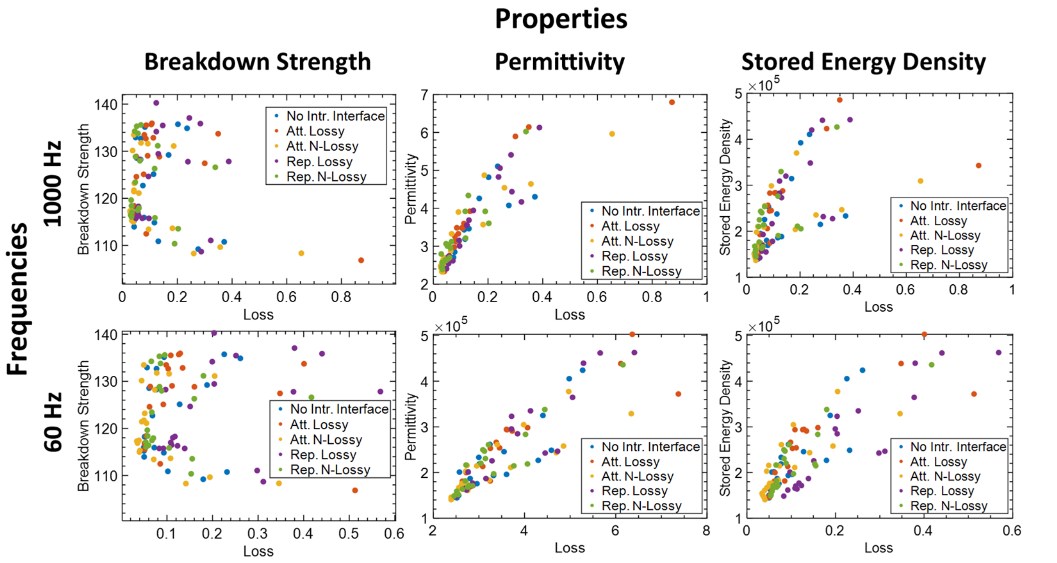

Figure S1. Nanodielectrics properties in the z direction based on the DOE designs at two frequencies 1000 Hz and 60 Hz with respect to intrinsic interface design choices.

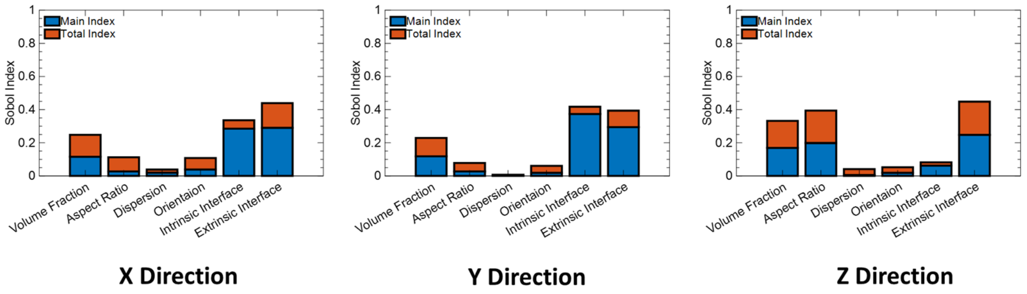

Figure S2. Global Sensitivity Analysis on the difference in permittivity at 1e-2 Hz and 1e7 Hz with respect to the mixed-variable design space.
